# High Spatiotemporal Resolution Monitoring of Water Body Dynamics in the Tibetan Plateau: An Innovative Method Based on Mixed Pixel Decomposition

**DOI:** 10.3390/s25041246

**Published:** 2025-02-18

**Authors:** Yuhang Jing, Zhenguo Niu

**Affiliations:** 1 Key Laboratory of Remote Sensing and Digital Earth, Aerospace Information Research Institute, Chinese Academy of Sciences, Beijing 100094, China; jingyuhang22@mails.ucas.ac.cn; 2 University of Chinese Academy of Sciences, Beijing 100049, China

**Keywords:** Tibetan Plateau, surface water, mixed pixel decomposition, abundance maps, wetland remote sensing

## Abstract

The Tibetan Plateau, known as the “Third Pole” and the “Water Tower of Asia”, has experienced significant changes in its surface water due to global warming. Accurately understanding and monitoring the spatiotemporal distribution of surface water is crucial for ecological conservation and the sustainable use of water resources. Among existing satellite data, the MODIS sensor stands out for its long time series and high temporal resolution, which make it advantageous for large-scale water body monitoring. However, its spatial resolution limitations hinder detailed monitoring. To address this, the present study proposes a dynamic endmember selection method based on phenological features, combined with mixed pixel decomposition techniques, to generate monthly water abundance maps of the Tibetan Plateau from 2000 to 2023. These maps precisely depict the interannual and seasonal variations in surface water, with an average accuracy of 95.3%. Compared to existing data products, the water abundance maps developed in this study provide better detail of surface water, while also benefiting from higher temporal resolution, enabling effective capture of dynamic water information. The dynamic monitoring of surface water on the Tibetan Plateau shows a year-on-year increase in water area, with an increasing fluctuation range. The surface water abundance products presented in this study not only provide more detailed information for the fine characterization of surface water but also offer a new technical approach and scientific basis for timely and accurate monitoring of surface water changes on the Tibetan Plateau.

## 1. Introduction

Surface water, as an essential component of the hydrological cycle, plays a critical role in maintaining the balance of water resources and the environment [1,2]. The Tibetan Plateau, often referred to as the “Water Tower of Asia”, not only accounts for more than half of China’s total number of lakes but also serves as the source for many major rivers [3]. The abundant water resources and unique ecosystems on the Plateau give rise to a complex and diverse hydrological cycle within a fragile ecological system [4]. However, the surface water resources of the Tibetan Plateau are highly sensitive to both regional and global climate change, making them crucial indicators of climatic and environmental shifts. Particularly under the backdrop of global warming, the rate of warming on the Tibetan Plateau is twice the global average, accelerating glacier melt and permafrost degradation. This process has disrupted the balance of surface water and increased the frequency of natural disasters such as ice avalanches and glacial lake outburst floods [5,6,7,8,9,10]. Therefore, monitoring the spatiotemporal variations in surface water on the Tibetan Plateau is vital for enhancing our understanding of climate change, aiding in disaster prevention, and supporting more effective management of surface water resources [11].

Remote sensing technology, due to its advantages of large-scale coverage, high efficiency, high spatial and temporal resolution, and low cost, has been widely used in the monitoring of surface water bodies. Prior to 2000, NOAA/VHRR and NOAA/AVHRR data were commonly used in water body monitoring studies [12,13]. After 2000, the advent of MODIS data gradually replaced AVHRR data [14], offering higher spatial resolution compared to NOAA satellites. However, the spatial resolution of MODIS data are still insufficient for fine-scale surface water monitoring. Landsat’s TM data, with higher spatial resolution, offers advantages in monitoring small water bodies; yet, its coarser temporal resolution limits its application in long-term surface water monitoring. Additionally, radar imagery, due to its strong penetration ability and all-weather observation capabilities, is often used to complement optical imagery [15,16]. However, its processing and analysis are complex and still require optimization.

To overcome the limitations of spatial resolution in remote sensing data, the mixed pixel decomposition method has emerged. This method decomposes mixed pixels, which consist of multiple land cover types, into different endmembers and calculates the proportion of each endmember within the pixel, thereby enabling the fine identification of complex land cover types. Xie Zhigang et al. used NDWI, MNDWI, SVM, and FCLS methods in combination with high-resolution Google imagery to study the precise extraction of surface water information from remote sensing images, evaluating the area accuracy of various extraction methods and selecting the optimal method for water body extraction [17]. Mayr et al. applied the mixed pixel decomposition model to propose a method for estimating global surface water coverage [18]. Feng et al. explored sub-pixel level information to monitor the changes in the surface water area of reservoirs [19]. Compared to traditional pixel-based classification methods, mixed pixel decomposition effectively improves the spatial resolution of remote sensing images and significantly enhances the accuracy of surface water extraction.

Many studies have been conducted on the dynamic monitoring of surface water bodies. At the global scale, Han Qianqian et al. generated a 250 m resolution, eight-day surface water product based on MODIS remote sensing imagery [20]. This product offers advantages in temporal resolution, but its coarse spatial resolution limits the accurate monitoring of surface water information. At the national scale, Li Yang et al. produced a 10 m resolution surface water product for 2016–2018 using Sentinel-1 satellite imagery [21], which offers higher spatial resolution but is limited by its short time span, failing to meet the needs of long-term surface water monitoring. While there have been some studies on the dynamic monitoring of surface water on the Tibetan Plateau, most research has focused on the region’s lakes. For example, Zhang Guoqing et al. explored the changes in lake area on the Tibetan Plateau from 1970 to 2013 using the NDWI index [4], and Mao D et al. assessed the spatiotemporal changes in lakes and glaciers on the Plateau between 1977 and 2014, analyzing the causes of lake variations [22]. However, research on the dynamic monitoring of surface water across the Tibetan Plateau is relatively limited, and existing surface water products for the region have not yet achieved long-term, high spatiotemporal resolution dynamic monitoring. Therefore, to enable dynamic monitoring of surface water on the Tibetan Plateau, this study aims to address two key issues: (1) developing a method based on mixed pixel decomposition suitable for long-term dynamic monitoring of surface water; (2) using this method to produce a set of long-term, high spatiotemporal resolution surface water products for the Tibetan Plateau. This research is of significant importance for timely and accurate monitoring of surface water changes in the Tibetan Plateau, enhancing our understanding of climate change, and providing essential support for the protection of the Plateau’s ecosystem and the sustainable management of its water resources.

## 2. Materials and Methods

### 2.1. Study Area

**Figure 1:**
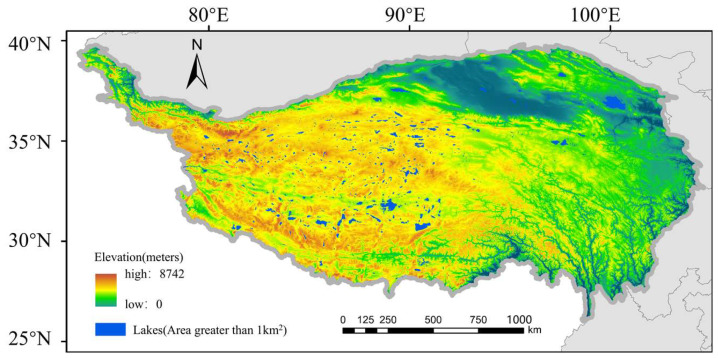
Study Area Overview.

The Tibetan Plateau, also known as the “Third Pole” and the “Water Tower of Asia”, has an average elevation exceeding 4000 m, making it the highest plateau in the world [23,24,25]. Due to its complex terrain and the influence of the Asian and Indian monsoons, the region has developed a unique plateau ecological environment that supports abundant water resources. As shown in Figure 1, the distribution of water resources on the Tibetan Plateau is influenced by factors such as precipitation, evaporation, glaciers, and permafrost, exhibiting a spatial pattern where the southeast is water-rich and the northwest is relatively arid [3,26]. Surface water resources in the region primarily consist of lakes (including salt lakes), glacial lakes, rivers, and their tributaries [11]. The Tibetan Plateau is the source of seven major rivers in Asia, including the Yangtze, Yellow, Nu, Lancang, Yarlung Zangbo, Ganges, and Indus Rivers [27]. The water systems on the Plateau can be divided into external and internal systems. The external systems, primarily located in the southeastern part of the Plateau, include the Yangtze, Yellow, and Lancang Rivers. The rivers in the northwest are mostly part of the internal water system. Compared to rivers in the external systems, those in the internal system are usually seasonal rivers, heavily influenced by climate change, and their water flow is unstable [3]. The Tibetan Plateau has a large number and concentration of lakes. These lakes make up half of the total lake area in China, with over 1000 lakes larger than 1 km^2^ [5,28]. The unique climate conditions and abundant water resources of the Tibetan Plateau make its response to climate change swift and significant.

### 2.2. Data Sources

#### 2.2.1. Study Data and Validation Data

Terra and Aqua are two important sun-synchronous polar-orbit satellites in the U.S. Earth Observing System (EOS) project, equipped with the Moderate-Resolution Imaging Spectroradiometer (MODIS). In this study, we used the MOD09A1 product from the MODIS Terra satellite, which provides 500 m surface reflectance data with an eight-day composite time interval, covering the period from 1 January 2000, to 31 December 2023. This product includes surface reflectance information from seven spectral bands. Since the core idea of mixed pixel decomposition is to treat the reflectance signal of a mixed pixel as a weighted sum of the spectral reflectance of different land cover types, reflectance data from multiple bands are needed to perform the decomposition. The rich band information makes this product particularly well-suited for the mixed pixel decomposition process. Additionally, the product has high temporal resolution and a long time span, making it ideal for long-term dynamic monitoring of surface water bodies.

We used the monthly water body product derived from Sentinel satellite data (China Water Cover Map-CWaC) [21], with a spatial resolution of 10 m, to evaluate the accuracy of the water abundance maps, calculate classification accuracy, and conduct intra-annual area trend analysis. The MODIS-based surface water product (Global Surface Water Extent Dataset, GSWED) [20], with a spatial resolution of 250 m and a temporal resolution of eight days, was also used for area trend analysis. Additionally, the JRC Global Surface Water Database product generated from Landsat data were employed for area trend analysis, with a spatial resolution of 30 m and a temporal resolution of monthly intervals [29].

#### 2.2.2. Auxiliary Data

The auxiliary data used in this study are as follows:

(1) SRTM DEM data, which is a digital elevation model (DEM) dataset with a spatial resolution of 90 m, produced by the Shuttle Radar Topography Mission (SRTM). In this study, areas with a slope value less than 5 degrees were selected to exclude the interference of mountain shadows in the water body extraction process.
(2) 500 m Resolution Normalized Difference Vegetation Index (NDVI) of the Tibetan Plateau (Loess Plateau SubCenter, National Earth System Science Data Center, National Science & Technology Infrastructure of China, http://loess.geodata.cn) (accessed on 18 October 2024). This dataset was used to assist in selecting vegetation and bare land endmembers.
(3) Tibetan Plateau Lake Dataset (V3.0) for lakes larger than 1 km^2^ [6,30]. This dataset was used to assist in selecting water body endmembers.
(4) Tibetan Plateau Snow Cover Dataset [31]. This dataset was used to assist in selecting snow endmembers.
(5) Annual classification data of the Maqu Wetland generated from Landsat time series. This dataset was used to explore the correlation between water body abundance and wetland areas [32].

## 3. Method

**Figure 2:**
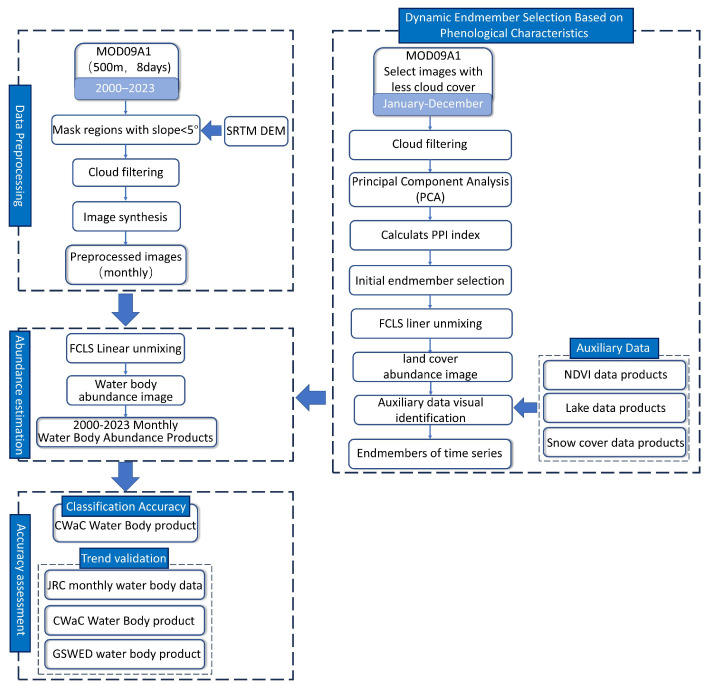
Workflow of Water Body Abundance Inversion on the Tibetan Plateau.

The process of generating the Tibetan Plateau water body abundance map consists of four steps (Figure 2), including preprocessing of surface reflectance data, dynamic endmember selection based on phenological characteristics, abundance inversion, and accuracy assessment.

### 3.1. Preprocessing of Surface Reflectance Data

First, the SRTM DEM data are used to calculate areas with a slope less than 5 degrees, which helps exclude the influence of mountain shadows. Then, based on the StateQA band, the number of cloud pixels is calculated, and cloud removal is performed to eliminate cloud interference, ensuring that cloud pixels do not affect the unmixing process.

### 3.2. Dynamic Endmember Selection Based on Phenological Characteristics

Since the spectral characteristics of endmembers for different land cover types vary over time, the spectral reflectance at a single point in time often fails to fully represent the true characteristics of the land cover. This study proposes a dynamic endmember selection method based on phenological characteristics. In traditional methods, endmembers are typically selected using Pixel Purity Index (PPI) and Principal Component Analysis (PCA). In this study, a secondary screening of endmembers is performed by combining auxiliary data with the traditional endmember selection methods. Additionally, spectral curves for the endmembers are generated on a monthly basis to capture the spectral variations in different land cover types under various seasonal or climatic conditions.

The specific steps are as follows: First, for each month, the image with the least cloud cover is selected. Principal Component Analysis (PCA) is then applied to reduce the dimensionality of the data. Next, the Pixel Purity Index (PPI) is used to initially select endmembers, from which the spectral reflectance of the endmembers is derived. Afterward, the Fully Constrained Least Squares (FCLS) Spectral Unmixing method is applied to perform the mixed pixel decomposition, resulting in an initial abundance map of land cover types. Once the initial abundance map is obtained, auxiliary data are used for visual interpretation to determine the land cover type of the endmembers and select pure endmembers. This step ensures that the selected endmembers are pure pixels and correctly represent the land cover type. In this study, five endmember categories were selected based on the land cover types of the Tibetan Plateau: snow, vegetation, soil, water bodies, and water body shadows. After generating the spectral reflectance curves of these land cover types for each month (from January to December), the endmember spectral reflectance curves based on phenological characteristics are obtained, laying the foundation for subsequent mixed pixel decomposition. The dynamic endmember selection method based on phenological characteristics employed in this study not only enables a more accurate identification of endmember types and the exclusion of non-pure pixels, but also facilitates a more refined characterization of the spectral reflectance of endmembers according to their phenological features. This, in turn, provides more precise endmember spectra for mixed pixel decomposition.

### 3.3. Abundance Inversion

Based on the extracted endmember spectral reflectance, linear unmixing is performed. We used the Fully Constrained Least Squares (FCLS) method, which is based on a linear mixing model. The FCLS method ensures that the abundance values of the mixed pixels remain within the range of 0 to 1 and that the sum of the abundance values for all land cover types equals 1.

### 3.4. Post-Processing of Abundance Images

Due to the presence of significant noise in the abundance images after mixed pixel decomposition, a thresholding method is used for post-processing to optimize the abundance results. First, the CWaC product for the period from January to December 2017 is used as a reference water sample area. The water body boundary from this product is used to determine the water body abundance threshold. Areas with abundance values greater than the threshold are considered valid water body regions. This threshold is then applied to the water body abundance image for post-processing, effectively removing noise from the abundance map.

### 3.5. Accuracy Assessment

The accuracy of the abundance images is evaluated using the 10 m resolution monthly water body product (CWaC) derived from Sentinel data. First, the maximum inundation map is generated from the Sentinel data, within which water body sample points are created, while non-water body sample points are generated in other areas. The 10 m resolution Sentinel water body distribution map is then aggregated into a 500 m resolution water body abundance image, which is raster-aligned with the water body abundance image produced in this study. Next, 7986 water body sample points are randomly selected from the water body abundance images for each month (from January to December), with a water to non-water sample point ratio of 10:1. We compared the abundance values obtained for each sample point from the CWaC product with those derived from the abundance image. The validation of the abundance image was carried out using various metrics, including commission rate, omission rate, classification accuracy, RSAE, and ME.

## 4. Results

### 4.1. Validation

First, the accuracy of the water body abundance image was assessed using the CWaC water body data product aggregated to a 500 m resolution. A total of 7986 validation sample points were selected, with a water-to-non-water sample point ratio of 10:1. These sample points were randomly distributed across the months of January to December 2017. The abundance values from the Sentinel water body data were used as ground truth and compared to the water body abundance images produced in this study. We defined commission as cases where the absolute difference between the two abundance values exceeded 0.5 and the difference was greater than 0, calculating the commission rate for the sample points. Omission was defined as cases where the absolute difference exceeded 0.5 and the difference was less than 0, leading to the calculation of the omission rate. The commission and omission rates are critical metrics for evaluating the performance of the model. Specifically, a lower commission rate indicates that fewer non-water areas are incorrectly classified as water bodies, while a lower omission rate suggests that the model is effective in identifying actual water bodies. For the remaining sample points, metrics such as root mean square error (RMSE), mean error (ME), and classification accuracy were calculated. These metrics are crucial for assessing the performance of our water abundance map. The ME provides insight into the systematic bias of the abundance map. A negative ME indicates that the predictions tend to underestimate the actual abundance, while a positive ME suggests overestimation. An ME close to zero indicates a balanced prediction with no consistent bias. Root Mean Square Error (RMSE) measures the average magnitude of the prediction errors, giving greater weight to larger errors due to the squaring of differences. RMSE is always non-negative, and a lower RMSE value indicates a better fit of the abundance map to the actual data. RMSE is particularly useful in contexts where large errors are undesirable, as it penalizes larger discrepancies more heavily than smaller ones. Classification accuracy was measured using the mean absolute error (MAE) to quantify the classification error of random points. MAE is defined as the average of the absolute errors between the actual abundance and the predicted abundance. Specifically, for n random points, the calculation formulas for ME, RMSE, and MAE are as follows:

(1) $$ME=\frac{1}{n}\sum_{i=1}^{n}\left({ŷ}_{i}-y_{i}\right)$$

(2) $$RMSE=\sqrt{\frac{1}{n}\sum_{i=1}^{n}{\left(y_{i}-{ŷ}_{i}\right)}^{2}}$$

(3) $$MAE=\frac{1}{n}\sum_{i=1}^{n}\left|y_{i}-{ŷ}_{i}\right|$$

In these formula, $y_{i}$ represents the actual abundance for the i-th point, while ${ŷ}_{i}$ represents the predicted abundance by the model for that point.

The formula for calculating classification accuracy *p* is:

(4) $$p=\left(1-MAE\right)\times 100\%$$

In the equation, p represents accuracy, while MAE denotes mean absolute error. The validation results are presented in Table 1.

**Figure 3:**
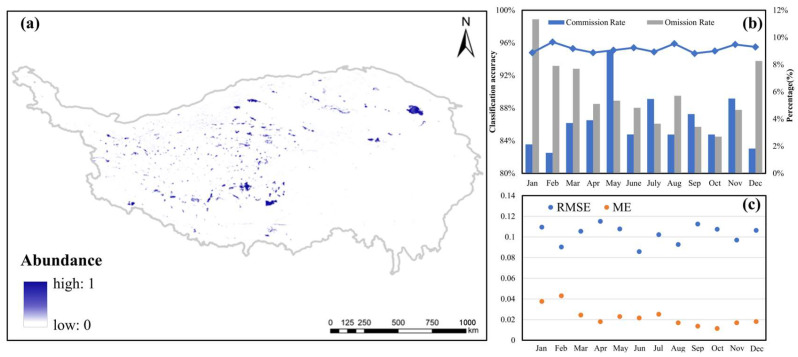
Abundance maps and validation results: (**a**) Abundance results for July 2017; (**b**) Distribution of classification accuracy, commission rate, and omission rate; (**c**) Scatter plot of RMSE and ME distribution.

An analysis of classification accuracy reveals good classification performance and stability. As shown in Figure 3b, the classification accuracy for each month exceeds 94%, with an average accuracy of 95.3% and a peak of 96.1%. This indicates that the hybrid pixel decomposition method employed is effective in extracting water body information, demonstrating consistent performance across different months. Consequently, it suggests that the abundance maps can accurately delineate water bodies for the majority of the time, thereby exhibiting a degree of reliability. The analysis of commission and omission rates across the months provides valuable insights into the model’s performance. For instance, the commission rates ranged from 1.51% to 11.33%, while the omission rates varied between 2.70% and 12.70%. Notably, both commission and omission rates are relatively high in January, which may be linked to the freezing period in the Tibetan Plateau region. During this time, the presence of lake ice interferes with the spectral signatures of surface water bodies, resulting in increased misclassifications. By analyzing these indicators, we can identify specific periods where the model may benefit from targeted improvements.

The root mean square error (RMSE) and mean error (ME) are analyzed, as shown in Figure 3c. the RMSE values for all months range from 0.08 to 0.11, indicating that the generated water body abundance images exhibit relatively small deviations from the actual data, with minimal inter-month variation. This further validates the reliability of the model. The results for mean error (ME) reflect the systematic bias in abundance estimation, with values ranging from 0.01 to 0.04, which also supports the accuracy of the abundance estimates.

### 4.2. Analysis of Area Changes over the Year

**Figure 4:**
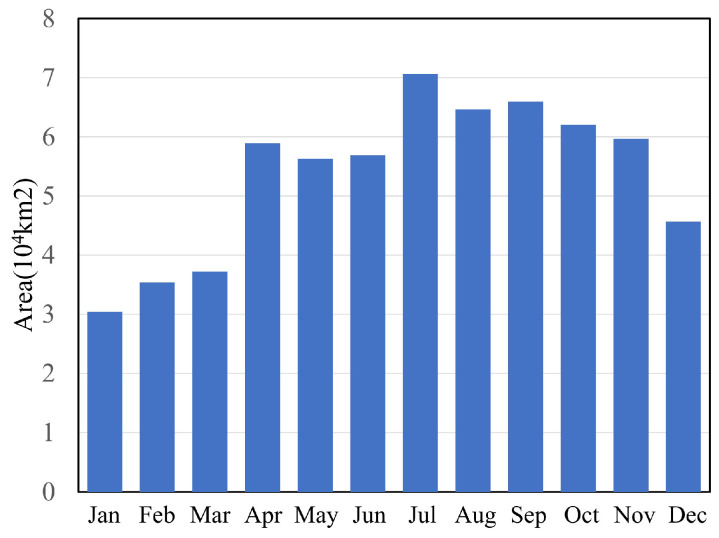
Analysis of Area Trend Over the Year.

A comparative analysis was conducted between the water body abundance map from 2017 and water body area data derived from other datasets collected during the same period, including GSWED, JRC, and CWaC water body products. As illustrated in Figure 4, it can be observed that the water body area indicated by the abundance map gradually increases from a relatively low value at the beginning of the year, reaching a peak in July, followed by a gradual decline.

**Figure 5:**
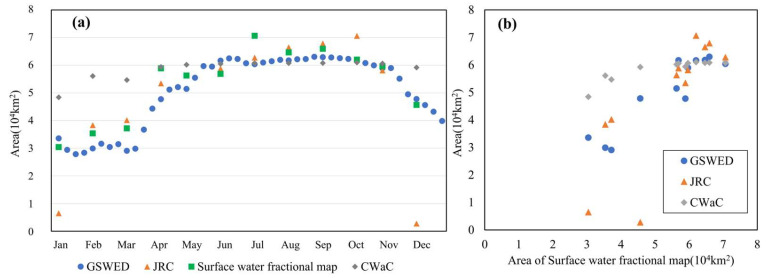
Comparison with Other Datasets: (**a**) Comparison of Area with Other Datasets; (**b**) Correlation of Abundance Map with Other Datasets.

Figure 5 illustrates the comparison of water body area between the 2017 Surface Water Fractional Map and other water body products (GSWED, JRC, and CWaC). As shown in the figure, the Surface Water Fractional Map exhibits a similar trend in water body area changes compared to the other products. During the dry season from January to March, the water body area in the Surface Water Fractional Map remains relatively stable at approximately 3 × 10^4^ km^2^. This value is closely aligned with that of GSWED but significantly higher than the water body areas reported by JRC and CWaC. Starting in April, as precipitation increases, the water body area in the Surface Water Fractional Map shows a marked increase, peaking during the wet season from May to September, fluctuating between 60,000 km^2^ and 70,000 km^2^. During this period, the changes in water body area for the Surface Water Fractional Map are comparable to those of GSWED and CWaC, although they are slightly higher than those reported by JRC. The differences between the products during the wet season are minimal, indicating a high degree of consistency. This suggests that the products are effective in capturing large-scale water body changes during periods of high water levels. As October approaches, a decline in water body area is observed across all products. The water body area in the Surface Water Fractional Map gradually decreases, reaching its lowest point of the year in December. In comparison with other water body products, the declining trend of the Surface Water Fractional Map aligns closely with that of GSWED and CWaC, while remaining slightly higher than that of JRC. This discrepancy may be attributed to differences in algorithms and data sources among the various products.

In summary, the Surface Water Fractional Map’s trends in water body area changes throughout 2017 are generally consistent with those of other water body products, particularly during both the high-water season and low-water season, where the variations are closely aligned. However, in terms of water body area, the Surface Water Fractional Map is more consistent with GSWED and CWaC, while it is slightly higher compared to JRC, indicating that the Surface Water Fractional Map is reliable in capturing changes in regional water body areas.

A correlation analysis between the abundance map and the water body areas from other datasets (Figure 5b) reveals that the correlation coefficient between the abundance map and CWaC is 0.87, while the correlation with GSWED is even higher at 0.92. The correlation coefficient between the abundance map and JRC data are 0.81. These coefficients indicate that the abundance map has the highest correlation with GSWED (0.92), suggesting a very high consistency in measuring water body area between these two datasets. This may be attributed to similarities in their data collection and processing methodologies.

Next, the abundance map shows a correlation coefficient of 0.87 with CWaC, which, although slightly lower than that with GSWED, still indicates a significant relationship. This further validates the consistency and reliability of the abundance map in estimating water body areas across different datasets. In contrast, the correlation with JRC is 0.81, which, while lower than the previous two, still suggests a good level of correlation. This discrepancy may reflect differences in methodologies between the JRC dataset and the abundance map, or could be due to variations in temporal coverage and spatial resolution. Overall, the abundance map demonstrates a high degree of correlation with all three water body area datasets, indicating its accuracy and consistency in estimating water body areas.

### 4.3. Interannual Area Change Trend Analysis

**Figure 6:**
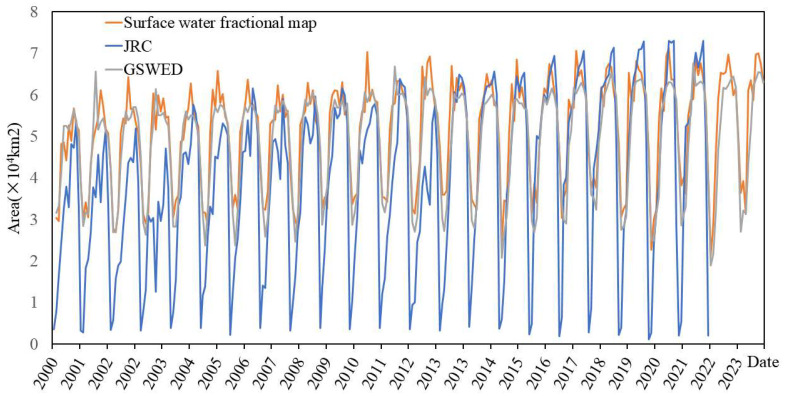
Interannual Area Change Diagram.

Figure 6 illustrates the area change trends of the water body abundance map product from 2000 to 2023. It is evident from the figure that there are significant interannual fluctuations in water body area, with the amplitude of these fluctuations gradually increasing over time. Specifically, throughout the entire period, the fluctuation range of water body area varies between 20,000 square kilometers and 70,000 square kilometers, exhibiting distinct periodic characteristics. This interannual variation indicates that water body area is influenced by seasonal factors as well as other environmental factors, while also displaying an upward trend over the entire time span.

The water body abundance map generated in this study exhibits a high degree of similarity to the JRC and GSWED water body products in terms of interannual area change trends and fluctuation amplitudes. All three datasets effectively capture the seasonal variations and interannual fluctuations in water body area, demonstrating a trend of gradual increase in water body area along with increasing volatility over the years. Specifically, the volatility of the abundance map is positioned between that of the JRC and GSWED products; it is more stable than the JRC product while exhibiting greater fluctuation than the GSWED product. Additionally, the increase in water body area of the abundance map falls between that of the JRC and GSWED products. From the perspective of water body area, the water body area in the abundance image is slightly larger than that of the JRC and GSWED water products before 2013. However, after 2013, due to a more significant growth trend in the JRC water body product, the area of the abundance image is slightly lower than that of the JRC product but remains higher than that of the GSWED product.

## 5. Discussion

### 5.1. Correlation Analysis with Other Water Body Products

**Figure 7:**
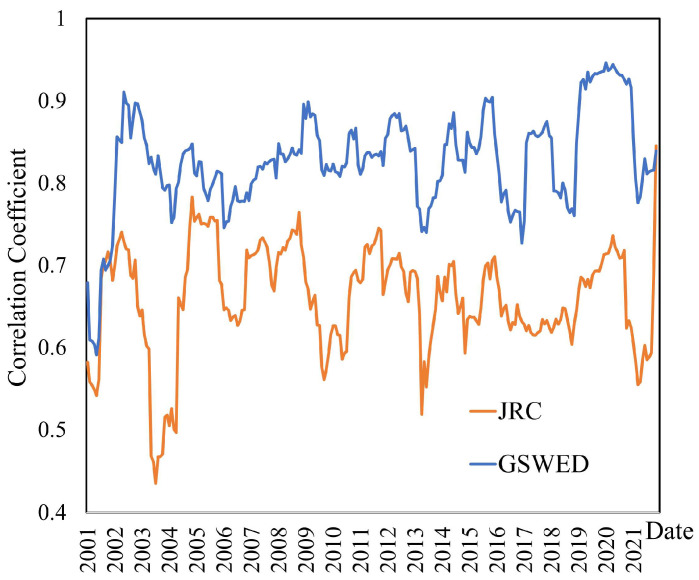
Correlation Analysis with JRC and GSWED Datasets.

We conducted a sliding window correlation analysis (with a window size of 12 months) to compare the correlation trends between the water body abundance map produced in this study and the JRC and GSWED datasets. As illustrated in Figure 7, the correlation coefficient between the water body abundance map and the GSWED dataset is generally high with minimal fluctuations, consistently remaining above 0.7. This indicates a strong consistency and high correlation between the two datasets. In contrast, the correlation coefficient with the JRC dataset is significantly lower and exhibits greater fluctuations, predominantly ranging between 0.3 and 0.7. This disparity in correlation can be attributed to the data sources utilized for the water body products. Both the abundance product and the GSWED water body product employ MODIS surface reflectance data as their source, which accounts for the higher correlation. Conversely, the JRC water body product relies on Landsat imagery, which has a lower temporal frequency and is unable to effectively capture dynamic water body changes, leading to a lower correlation with the water body abundance map produced in this study. The analysis indicates that the water body abundance map generated in this study exhibits a certain degree of correlation with both the GSWED and JRC datasets. These findings provide important insights into the applicability of different datasets for water body abundance monitoring applications.

### 5.2. Identification Results of Small Water Bodies from Abundance Map

**Figure 8:**
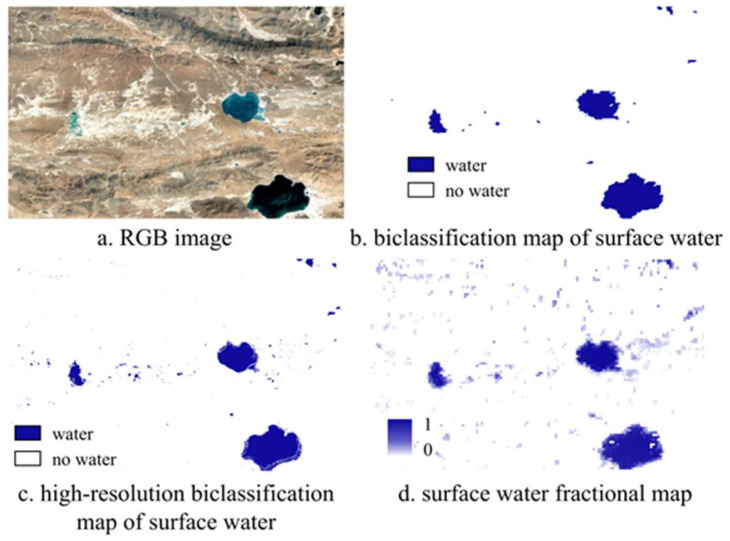
Identification Results of Small Water Bodies.

The water body abundance map effectively presents continuous variations in moisture levels through abundance values. Due to the spatial heterogeneity of water bodies, the boundaries of water areas are often unclear, and soil moisture content can be high. The use of the water body abundance map allows for a more accurate recording and expression of these detailed features. As illustrated below in Figure 8, it is evident that the GSWED product, which relies on traditional binary classification for water body extraction, delineates water areas through clear land-water boundaries. However, it struggles to accurately capture changes in small water bodies and the blurred boundaries of water areas, resulting in a relatively coarse identification outcome. In contrast, the water body abundance map produced in this study can more delicately represent the changes in small water bodies and complex water body boundaries. Moreover, it approaches the high-resolution CWaC water body product, allowing for observation of the gradient changes in water abundance through variations in grayscale values. This representation highlights the gradient variations in moisture content near the boundaries, providing a more nuanced understanding of small water bodies and their dynamics.

### 5.3. Identification Results of Linear Water Bodies

The water body abundance map demonstrates a significant advantage in the identification of linear water bodies. In monitoring linear water bodies, traditional binary classification methods, such as those used in the GSWED water body product, often struggle to accurately capture subtle water distribution, particularly in narrow waterways like rivers and streams. In contrast, the water body abundance map allows for a more detailed representation of moisture content across different areas, enabling high-precision identification and extraction of linear water bodies. This capability is crucial for effectively monitoring and managing these essential aquatic ecosystems, as it provides a clearer understanding of their spatial extent and dynamics.

**Figure 9:**
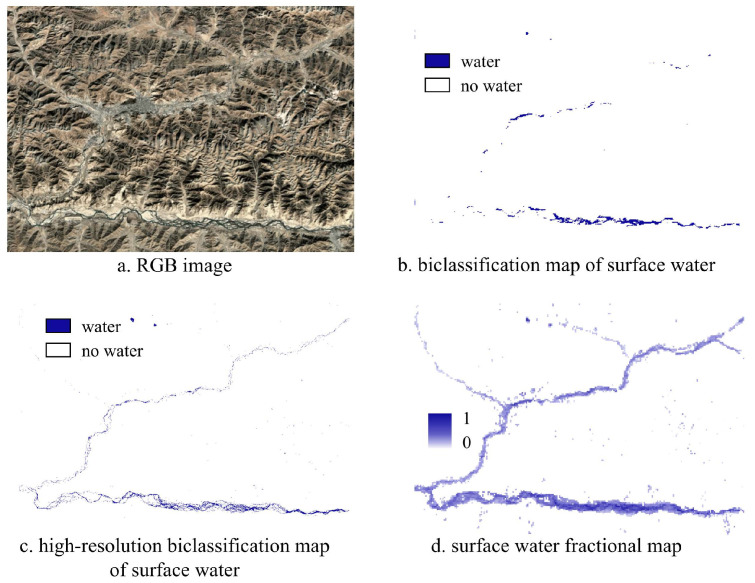
Identification of Linear Water Bodies.

As shown in Figure 9, the area contains multiple linear water bodies, including several rivers. The water body image produced by the GSWED product, which relies on a simple classification of water and non-water areas, demonstrates some effectiveness in identifying broader water bodies. However, it performs poorly in extracting narrow rivers and tributaries, often leading to omissions and misclassifications. In contrast, the water body abundance map generated in this study provides a more detailed representation of the distribution of linear water bodies. The figure clearly depicts the winding course of the rivers, effectively extracting information about the main river channel, while also offering a more nuanced representation of its tributaries and adjacent wetlands. This map highlights the differences in moisture abundance across various regions. Furthermore, the high-resolution CWaC water product extraction further illustrates the detailed distribution of linear water bodies. When compared to the results of the water body abundance map, both products exhibit consistency in finer details, providing very precise spatial localization. However, the water body abundance map conveys variations in water content with greater nuance. Thus, the water body abundance map not only accurately characterizes the distribution of river systems but also reveals the differences in moisture content across different regions. This provides a scientific basis and support for wetland conservation and river management efforts. Moreover, this detailed representation is crucial for a deeper understanding and management of regional water resources.

### 5.4. Potential of Abundance Maps in Wetland Classification

**Figure 10:**
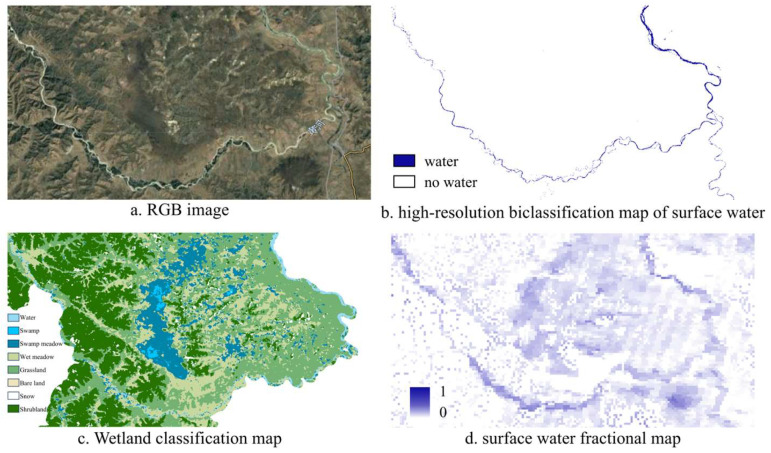
Potential of Abundance Maps in Wetland Classification.

The primary advantage of the water body abundance map lies in its ability to reveal subtle variations in moisture content within a region, which is particularly important for wetland monitoring. Wetland areas often lack distinct boundaries, yet they typically exhibit high soil moisture content. The water body abundance map effectively captures and expresses these characteristics. As shown in Figure 10 below, the area in question is a floodplain wetland that cannot be adequately monitored using high-resolution binary classification water body maps. However, the water body abundance map successfully characterizes the distribution of water bodies in floodplains, marshes, and other wetland types. By analyzing the abundance values, we can further detail the abundance ranges of different wetland types, such as floodplains and marshes. This, in turn, allows for a deeper understanding of the spatial distribution of water content within wetlands, providing a scientific basis for wetland conservation and management.

### 5.5. Research Limitation

This study focused on the Tibetan Plateau region, where the proposed endmember selection method based on phenological characteristics demonstrated effective water body extraction results. However, factors such as climate, topography, land use, and hydrological conditions in different geographic areas may significantly influence the spectral characteristics of water bodies. Consequently, the limitation of this method lies in the necessity to conduct multiple selections of endmembers for water bodies and other land cover types based on the specific characteristics of different regions. This requirement results in reduced efficiency when performing water body extraction at larger scales, thereby warranting caution regarding the generalizability of the research findings. Future studies should aim to enhance the applicability of this method across broader geographic ranges to validate and extend our findings, ultimately leading to a more comprehensive understanding of the mechanisms underlying changes in water body characteristics.

## 6. Conclusions

This study utilized a mixed pixel decomposition method to overcome the spatial resolution limitations of MODIS satellite data, resulting in a surface water body abundance product that meets high temporal and spatial resolution requirements. A dataset of 7986 sample points was selected for validation, and the results indicate that the overall classification accuracy of this product exceeds 95.3%, reaching as high as 96.1%. The product demonstrated good classification performance in monitoring small water body boundaries and linear water bodies, and also showed a certain level of capability in categorizing wetland types such as floodplains. Additionally, the analysis revealed that the surface water area in the Tibetan Plateau exhibits a cyclical and upward trend. In summary, the water body abundance product developed in this study enhances the capability for dynamic monitoring of surface water bodies and holds significant potential for future applications in wetland classification.

Future research can further optimize the mixed pixel decomposition method, focusing on sub-pixel localization based on abundance images to enhance the spatial resolution of water body products. This is particularly important in the context of global climate change, as long-term monitoring and analysis of surface water dynamics across different regions and seasonal variations will aid in better understanding the spatial and temporal patterns of water resources. Additionally, integrating machine learning and artificial intelligence technologies to develop automated water body monitoring systems represents a significant research direction. Such advancements will not only improve the monitoring capabilities of surface water bodies but also provide more accurate and timely data for environmental protection and water resource management.

## Figures and Tables

**Table 1:** Validation Results of the Abundance Image.

	January	February	March	April	May	June	July	August	September	October	November	December
Classification Accuracy	94.8%	96.1%	95.3%	94.8%	95.1%	95.4%	94.9%	95.9%	94.7%	95.0%	95.8%	95.5%
RMSE	0.11	0.09	0.10	0.11	0.10	0.08	0.10	0.09	0.11	0.10	0.09	0.10
ME	0.03	0.04	0.02	0.02	0.02	0.02	0.03	0.02	0.01	0.01	0.02	0.02
commission rate	11.33%	1.51%	3.70%	3.91%	9.06%	2.86%	5.47%	2.85%	4.35%	2.86%	5.50%	1.82%
omission rate	12.70%	7.90%	7.69%	5.11%	5.35%	4.82%	3.65%	5.71%	3.42%	2.70%	4.67%	8.26%

## Data Availability

The raw data supporting the conclusions of this article will be made available by the authors on request.
